# Study on Improving the Precise Machinability of Single Crystal SiC by an Ultrasonic-Assisted Hybrid Process

**DOI:** 10.3390/ma14237320

**Published:** 2021-11-30

**Authors:** Dong Shi, Tianchen Zhao, Tengfei Ma, Jinping Pan

**Affiliations:** 1 College of Mechanical Engineering, Quzhou University, Quzhou 324000, China; 36097@qzc.edu.cn (T.Z.); 36105@qzc.edu.cn (T.M.); 2 Zhejiang Haina Semiconductor Co., Ltd., Quzhou 324300, China; panjinping@hainasemi.cn

**Keywords:** SiC devices, SiC wafer, precise machinability, hybrid process, ultrasonic-assisted lapping, ultrasonic-assisted CMP

## Abstract

Silicon carbide (SiC) devices have become one of the key research directions in the field of power electronics. However, due to the limitation of the SiC wafer growth process and processing capacity, SiC devices, such as SiC MOSFET (Metal-oxide-semiconductor Field-effect Transistor), are facing the problems of high cost and unsatisfied performance. To improve the precise machinability of single-crystal SiC wafer, this paper proposed a new hybrid process. Firstly, we developed an ultrasonic vibration-assisted device, by which ultrasonic-assisted lapping and ultrasonic-assisted CMP (chemical mechanical polishing) for SiC wafer were fulfilled. Secondly, a novel three-step ultrasonic-assisted precise machining route was proposed. In the first step, ultrasonic lapping using a cast iron disc was conducted, which quickly removed large surface damages with a high MRR (material removal rate) of 10.93 μm/min. In the second step, ultrasonic lapping using a copper disc was conducted, which reduced the residual surface defects with a high MRR of 6.11 μm/min. In the third step, ultrasonic CMP using a polyurethane pad was conducted, which achieved a smooth and less damaged surface with an MRR of 1.44 μm/h. These results suggest that the ultrasonic-assisted hybrid process can improve the precise machinability of SiC, which will hopefully achieve high-efficiency and ultra-precision machining.

## 1. Introduction

In recent years, silicon carbide (SiC) devices have become one of the key research directions in the field of power electronics. Due to their excellent performance parameters, such as small unit conduction resistance, high-frequency switching, high-temperature operation, high pressure, and radiation resistance, many countries in the world are making efforts to develop SiC devices. They are expected to be used in frequency converters in various industries as soon as possible to replace the existing silicon devices [1]. For example, SiC MOSFET (Metal-oxide-semiconductor Field-effect Transistor) has been the focus of numerous investigations. MOSFET is a key element in modern microelectronics, which is widely used in highly integrated CMOS (Complementary Metal Oxide Semiconductor) and high-power devices. Among the more than 200 possibilities of SiC crystallographic structures, the so-called polytypes, the most commonly used for device fabrication have traditionally been the 3 C (cubic), 6 H and 4 H (hexagonal) polytypes [2]. The defects on the substrate surface could be replicated into the epilayer, thus surface preparation of the substrate wafer will play a critical role in electronic integration fabrication. Si-face (0001) is more difficult to be removed than C-face. Compared with C-face, Si-face is considered to be more useful for device fabrication or as a substrate for epitaxial film growth [3]. Fabricating SiC devices required large amounts of SiC semiconductor wafers. However, due to the limitation of the SiC wafer growth process and processing capacity, the current carrying capacity of SiC MOSFET is much lower than that of Si IGBT [4,5]. Another factor restricting the current carrying capacity of SiC MOSFET is the cost of fabricating a large-sized SiC wafer. Increasing the chip area is the main method to improve the current carrying capacity. At present, there are common defects at the SiC substrate for epitaxy. Forcibly increasing the chip area will lead to a sharp decline in yield and soaring price [6,7]. Therefore, the high-efficiency and ultraprecise polishing technology of single-crystal SiC wafers determine the wide application and deep development of silicon carbide devices.

At present, chemical mechanical polishing (CMP) is a necessary technology for semiconductor wafer finishing. However, due to the high hardness and inertia of single-crystal SiC, the material removal rate (MRR) of CMP is very low. Therefore, domestic and foreign scholars in the field of precise machining have put forward many hybrid machining methods by introducing the external energy field. Electrochemical mechanical polishing (ECMP) [8,9], plasma-assisted polishing [10,11], UV (ultraviolet) photocatalytic-assisted polishing [12,13], laser-induced assisted polishing [14,15], and Fenton oxidation-assisted polishing [16] were included. In essence, these methods improved the oxidation corrosion performance of SiC by different external energies, which made the material easy to be removed. However, most of the methods have shortcomings. Except for ECMP and UV-assisted polishing, which had MRRs of one micron per hour, other methods still had low MRR. The UV photocatalytic method has poor stability and UV light is harmful to the human body [17]. Owing to the uneven oxidation of the SiC surface, with ECMP it was difficult to obtain an atomically smooth surface [18]. Etch pits were generated under a higher oxidation rate, which significantly increased the surface roughness. The surface quality after ECMP did not satisfy the requirements for electronic devices [19]. It is well-known that ultrasonic-assisted technology has beneficial effects in fabrication [20]. Our previous paper has preliminarily investigated the beneficial effects and the mechanisms in machining SiC by ultrasonic vibration-assisted CMP after ultrasonic-assisted lapping [21]. However, except for that research, using ultrasonic-assisted CMP combined with ultrasonic-assisted lapping to further improve the precise machinability of single-crystal SiC has hardly been reported.

Therefore, this paper proposed a new hybrid process, which combined ultrasonic-assisted lapping with ultrasonic-assisted CMP, to further improve the precise machinability of single-crystal SiC. Firstly, an ultrasonic vibration-assisted device was designed, where the key part of an ultrasonic stepped horn was emphatically introduced. Secondly, the effects of ultrasonic vibrated amplitude and lapping disc material on the machinability of SiC lapping were experimentally investigated. Simultaneously, the influence of ultrasonic vibration on CMP were implied by comparative experiments. Finally, the precise machining route of SiC was designed based on the former experimental results. After the arranged experiments, an ideal surface with improved MRR was achieved.

## 2. Experimental Procedures

Firstly, individual ultrasonic-assisted lapping and ultrasonic-assisted CMP experiments were conducted. The effects of vibration amplitude and lapping disc material on the machining performance of single-crystal SiC were both investigated. Si-face 6H-SiC (±0.5° off axis) with a size of 2 inches was provided by Tankeblue semiconductor (Beijing, China). Secondly, the ultrasonic-assisted hybrid machining route was designed. Ultrasonic lapping combined with ultrasonic CMP was performed to investigate the precise machinability of SiC. The experimental setup consists of the precise machine BNI62 and an ultrasonic vibration-assisted device. BNI62 was purchased from BN Technology Corporation in Tokyo, Japan. The ultrasonic device was designed and manufactured by ourselves. The consumable materials in lapping mainly include diamond slurry and the lapping disc. The materials of the three lapping discs are cast iron, copper and aluminum. In CMP, the consumable materials and tools are mainly colloidal silica polishing liquid, polyurethane polishing pad, hydrogen peroxide oxidant (6 wt%) and potassium hydroxide catalyst (0.6 wt%). During the experiments, a precise electronic scale with a resolution of 0.1 mg, and an ultra-depth three-dimensional microscope (OLYMPUS DSX500, Tokyo, Japan) were adopted to obtain the machining efficiency and surface quality. This microscope can build 3D images with full focus and high precision. Even the samples with large concave and convex morphology or samples with height difference surface morphology can be clearly observed. During the design and establishment of the ultrasonic vibration-assisted device, a laser Doppler vibrometer (MetroLaser VibroMet^TM^ 500V, Laguna Hills, CA, USA) and an impedance analyzer (Wayne Kerr 6500B, London, UK) were used to detect the ultrasonic amplitude and ultrasonic frequency, respectively.

## 3. Establishing the Ultrasonic Vibration-Assisted Device

### 3.1. Facility Design

**Figure 1:**
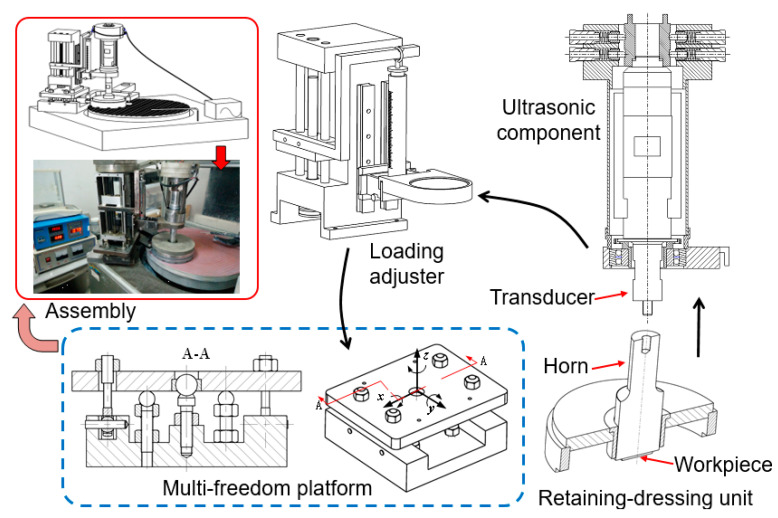
Experimental setup of ultrasonic vibration-assisted composition process.

Figure 1 shows the self-designed and self-established ultrasonic vibration-assisted device. It is mainly composed of four parts, which are the retaining-dressing unit, ultrasonic component, loading adjuster and multifreedom platform. All the parts are assembled together according to the arrows and then placed on the precise machine, BNI62. The ultrasonic component provides ultrasonic vibration via a digital ultrasonic power. The loading adjuster is used to provide the lapping or polishing pressure. The retaining-dressing unit is used to ensure the only rotational movement of the workpiece. Additionally, it has the dressing function for the lapping disc or polishing pad. The multifreedom platform is used to guarantee the positional relationship between the workpiece and machine rotational plate. It has the universal regulating function. Based on this device, the main working process in ultrasonic lapping and ultrasonic CMP is as follows:

(1) The workpiece is mounted on the stepped horn through paraffin wax.
(2) The horn is installed on the ultrasonic transducer through a set screw.
(3) Adjust the rocker arm of the machine and place the retaining ring therein.
(4) Adjust the multifreedom platform, place the horn and workpiece on the rotational plate of BNI62 machine through the retaining-dressing unit.
(5) Adjust the loading adjuster, set the lapping or polishing pressure, supply the processing fluid, set the speed, and start the machine.

### 3.2. Design of the Stepped Horn and Its Performance Test

The stepped horn was designed according to the longitudinal vibration wave equation. The specific design process refers to a previous study, where the amplification coefficient of the stepped horn was determined to be 0.35 by means of theoretical calculation shown in Equation (1) and numerical analysis of harmonic response [22].

(1) $$K=\frac{S_{\mathrm{out}}}{S_{\mathrm{input}}}$$

where, $S_{\mathrm{input}}$, $S_{\mathrm{out}}$ are the cross-sectional areas of the input and output ends in the stepped horn, respectively.

**Figure 2:**
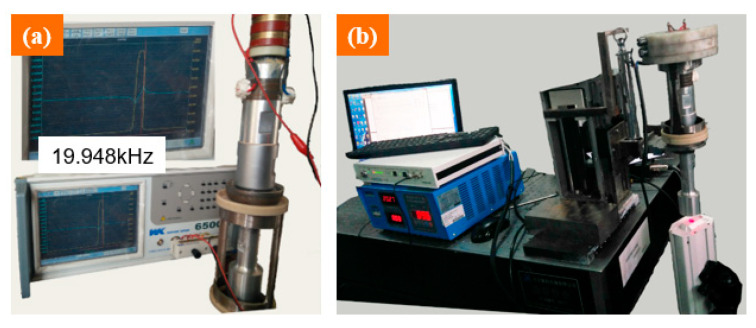
Performance test of the stepped horn: (**a**) actual resonance frequency; (**b**) actual vibration amplitude detection.

According to the designed data, the stepped horn was manufactured. Its actual performance was tested by an impedance analyzer and laser Doppler vibrometer, as shown in Figure 2. According to the impedance analyzer, a resonant frequency of 19.948 kHz was obtained, as shown in Figure 2a. Compared with the target value of 20 kHz, the error was within the accepted range of ±500 Hz. This frequency was within the working range of the digital ultrasonic power supply, which suggested that the horn could produce resonance. According to the laser Doppler vibrometer, the actual ultrasonic parameters including frequency and amplitude under different powers were obtained. The designed amplitudes and the actual values are all listed in Table 1, which were used to verify the consistency of design and manufacture. The rated power (P) of the digital ultrasonic power supply is 2 kW. To test the actual performance of the manufactured horn, the power was supplied from 40% P to 90% P.

From Table 1, it can be seen that vibrated amplitude increases as the power increases, but the corresponding frequency tends to decrease slightly. In addition, in order to verify whether the actual scale coefficient of the horn is consistent with the designed value of 0.35, the theoretically computed amplitudes were listed in the column of designed output. Additionally, the actual amplitudes were listed in the third column. By comparing the values of the two columns, it can be found that the actual values are slightly smaller than the corresponding theoretical values because of the actual energy loss. However, the actual results are still consistent with the designed conditions, which suggests that the manufactured horn can be used to conduct subsequent ultrasonic-assisted experiments.

## 4. Results and Discussion

### 4.1. Effects of the Vibrated Amplitude

**Figure 3:**
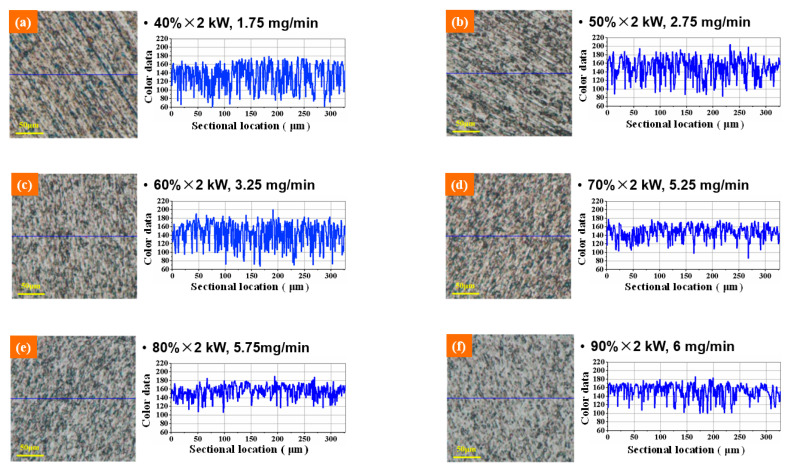
Optical images and sectional surface profiles after ultrasonic vibration-assisted lapping under different amplitudes: (**a**) 40% × 2 kW; (**b**) 50% × 2 kW; (**c**) 60% × 2 kW; (**d**) 70% × 2 kW; (**e**) 80% × 2 kW; (**f**) 90% × 2 kW.

Based on the ultrasonic-assisted lapping experiments with different amplitudes, the effects of amplitude on the machining performance of ultrasonic-assisted lapping were analyzed. According to the relationship between amplitude and ultrasonic power in Table 1, six kinds of ultrasonic powers were set for comparative experiments, which were 40% × 2 kW, 50% × 2 kW, 60% × 2 kW, 70% × 2 kW, 80% × 2 kW and 90% × 2 kW, respectively. Other main technological conditions, including normal pressure (P), lapping disc rotational speed (n), diamond abrasive particle size (D), lapping time (t), slurry supplying speed (v), and diamond abrasive concentration (C), are all listed in Table 2. In the table, rpm refers to revolutions per minute and wt% is mass percent. The micromorphology and color data of the lapped surface were obtained by the ultra-depth three-dimensional microscope, as shown in Figure 3. From Figure 3a–f, the left part is the microscopic defects of the SiC surface after lapping. The right curve is the color data of the surface sectional profile, which were used to analyze the line roughness of the machined surface. The vertical axis of the “color data” means the RGB value. Lower RGB suggests a concave surface and higher RGB suggests a convex surface. When the RGB values have large fluctuation, it is demonstrated that the surface roughness is large. On the contrary, a flat curve with little fluctuation implies a little surface roughness. Figure 3 shows that the fluctuations in color data curves in the last three decrease significantly compared with the former three. Lower fluctuation suggests that the surface roughness decreased. Figure 3a,b displays that obvious continuous scratches have formed on the surface. Continuous scratches easily produce deep subsurface damages. This can explain why the former three with relatively small power, namely relatively small amplitude, have relatively large, fluctuated curves. In addition to analyzing the above surface creation results, the material removal rates of the lapping experiments were calculated according to Equation (2). From low power to high power, the corresponding material removal rates were 1.75 mg/min, 2.75 mg/min, 3.25 mg/min, 5.25 mg/min, 5.75 mg/min and 6 mg/min, respectively. To sum up, in the range of ultrasonic amplitude, increasing amplitude can increase the processing efficiency of single-crystal SiC. When the amplitude increases to 2 μm or a higher value, namely the power is greater than or equal to 70% × 2 kW, surface defects and damages are also reduced. These results imply that ultrasonic-assisted lapping with a proper amplitude can comprehensively improve machining efficiency and surface quality. Less surface damages are beneficial to reduce the subsequent process time. Therefore, ultrasonic assistance promotes achieving a finishing surface with a higher MRR.

(2) $${\mathrm{MRR}}_{1}=\Delta m/t$$

where, Δm is the mass change of SiC wafer before and after lapping; t is the lapping time.

**Figure 4:**
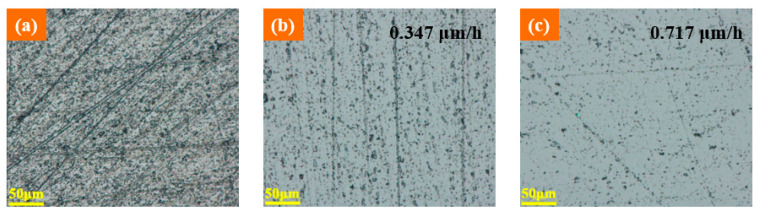
Influence of ultrasonic vibration on CMP: (**a**) optical image after common lapping; (**b**) optical image after common CMP after common lapping; (**c**) optical image after ultrasonic CMP after common lapping.

Based on the above results, ultrasonic-vibrated chemical mechanical polishing was also investigated by comparative experiments. Conventional CMP and ultrasonic-assisted CMP were performed separately on the conventional lapped wafers for 1 h. The main polishing conditions were a vibration amplitude of 2 μm, rotary speed of 40 rpm, load pressure of 60 kPa, and slurry feeding rate of 25 mL/min. Figure 4 displays the experimental results, which are used to show the effects of ultrasonic vibration on CMP. The surface micromorphologies were obtained by ultra-depth three-dimensional microscope and the machining efficiency was calculated by Equation (3). As shown in Figure 4b,c, the surface defects after ultrasonic-assisted polishing are significantly reduced compared with conventional polishing. The material removal rate of ultrasonic-assisted polishing was 0.717 μm/h, which was higher than the 0.347 μm/h of conventional polishing. It can be inferred that ultrasonic-assisted polishing can improve efficiency, thus quickly removing the surface defects and damages produced by the former lapping.

(3) $${\mathrm{MRR}}_{2}=\frac{\Delta h}{t}=\frac{\Delta m/\rho A}{t}$$

where, Δh is the thickness change of SiC wafer before and after lapping; Δm is the mass change of SiC wafer before and after lapping, weighed by a precise electronic scale; ρ is the density of SiC wafer; A is machining area of SiC wafer; t is the polishing time.

### 4.2. Effects of Lapping Disc Material

**Figure 5:**
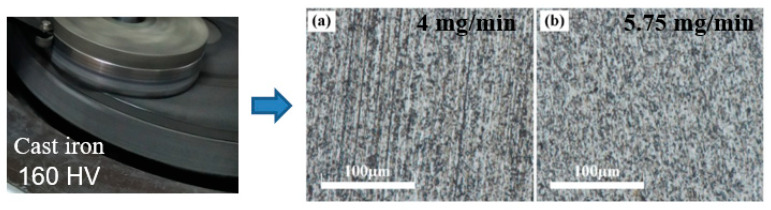
Optical images after lapping using cast iron pad: (**a**) common lapping; (**b**) ultrasonic-assisted lapping.

**Figure 6:**
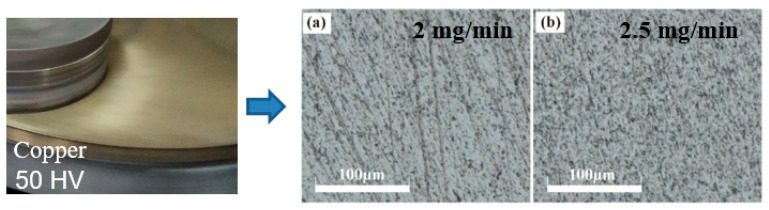
Optical images after lapping using copper disc: (**a**) common lapping; (**b**) ultrasonic-assisted lapping.

**Figure 7:**
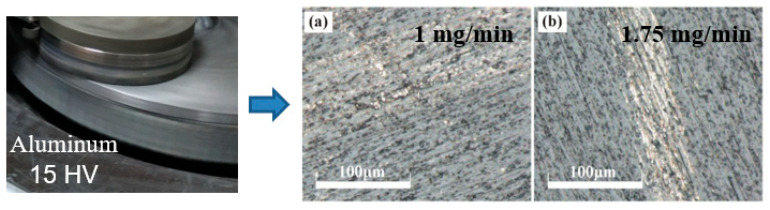
Optical images after lapping using aluminum disc: (**a**) common lapping; (**b**) ultrasonic-assisted lapping.

By conventional lapping and ultrasonic-assisted lapping, Figure 5, Figure 6, Figure 7 display the experimental results using three lapping discs. In Figure 5b and Figure 6b, it can be found that by using cast iron disc and copper disc after ultrasonic-assisted lapping, the surface defects of polished SiC were mainly pits. While the surface defects include scratches in traditional lapping as shown in Figure 5a and Figure 6a. The pits were caused by the brittle removal of single-crystal SiC due to the fact that under the action of ultrasonic vibration, abrasive particles will impact the SiC surface. Comparatively speaking, surface defects using a cast iron lapping disc are more serious than that using a copper lapping disc. The above experimental results suggest that the material removal mode of single-crystal SiC will transform from brittleness to ductility when decreasing lapping disc hardness. Abrasives have a yield effect on the lapping disc, and [23] has reported this. That is to say, the abrasives are easily pressed into the lapping disc with lower material hardness under the same pressure. The relationship between the pressing depth, d, of abrasive particles in the lapping disc, the normal force of abrasive particles and the hardness of the lapping disc are given in Equation (4). It shows that the pressing depth of abrasive grains in the lapping disc is inversely proportional to the hardness of the lapping disc. Therefore, using a lower hardness lapping disc will reduce the machining pressure, which can reduce the surface damages. However, the machined surface quality using an aluminum lapping disc, as seen in Figure 7, is worse than that using a copper disc with a larger hardness of 50 HV. The surfaces are characterized by more scratches and serious pits. Besides, surface defects have little difference between the traditional lapping and ultrasonic lapping when using an aluminum lapping disc. Based on the yield effect, it can be inferred that the pressed depth of abrasives into the disc were too large when using the aluminum disc. The large pressed depth of abrasives made the gap between the SiC and lapping disc become smaller. Therefore, large abrasives, other particles and impurities in the gap cannot be discharged in time, or direct dry friction forms between the SiC and lapping disc. These actions will damage the machined surface. To conclude, yield effect can reduce the mechanical effects of abrasives on SiC, namely decreasing the material removal rate, and it can also decrease the surface defects. Therefore, the choice of lapping disc material and processing pressure needed to be balanced and coordinated according to different technological purposes.

(4) $$d\propto \sqrt{\frac{P}{H}}$$

where, *P* is the lapping pressure applied on the workpiece; *H* is the hardness of the lapping disc.

### 4.3. Effects of Vibrated Lapping Followed by Vibrated CMP

Ultrasonic vibration can improve the machinability of single-crystal SiC in separate lapping and CMP. At the same time, the hardness of lapping disc has important effects on the surface quality and processing efficiency. Based on these results, the ultrasonic vibration-assisted manufacturing route for single-crystal SiC was proposed. The first step was ultrasonic lapping using a cast iron disc, the second step was ultrasonic lapping using a copper disc, and the third step was ultrasonic-assisted CMP using a polyurethane pad. The main conditions in the processing route are provided in Table 3.

**Figure 8:**
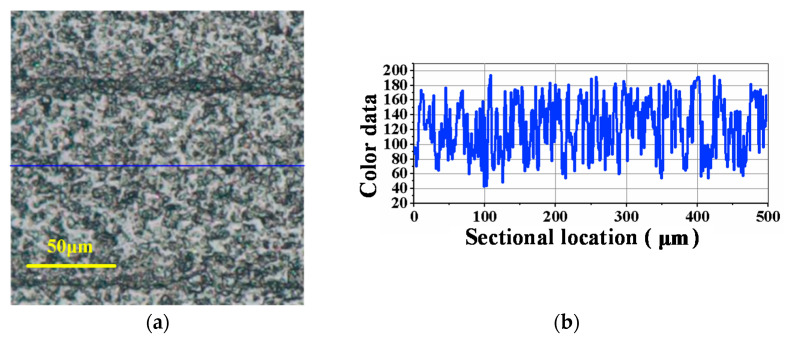
Received SiC surface before single-sided lapping: (**a**) Surface defects; (**b**) Color data of the sectional profile.

**Figure 9:**
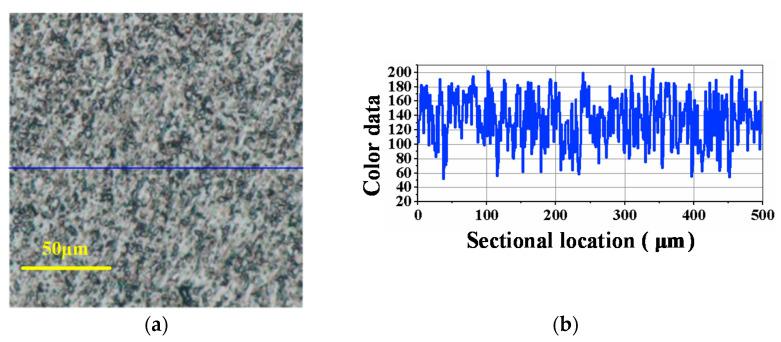
Optical image and sectional surface profile after ultrasonic-assisted lapping using cast iron disc (10.93 μm/min): (**a**) Surface defects; (**b**) Color data of the sectional profile.

**Figure 10:**
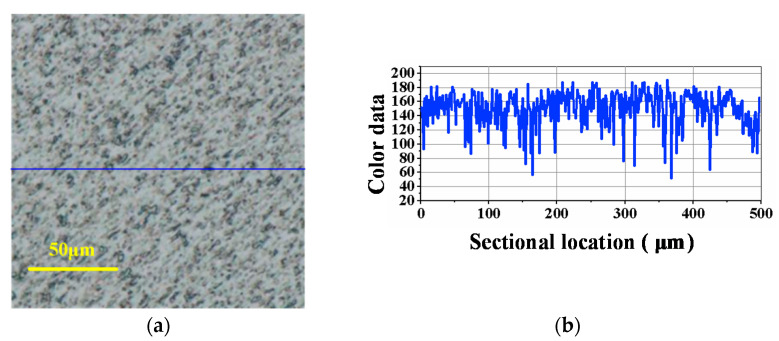
Optical image and sectional surface profile after ultrasonic-assisted lapping using copper disc (6.11 μm/min): (**a**) Surface defects; (**b**) Color data of the sectional profile.

**Figure 11:**
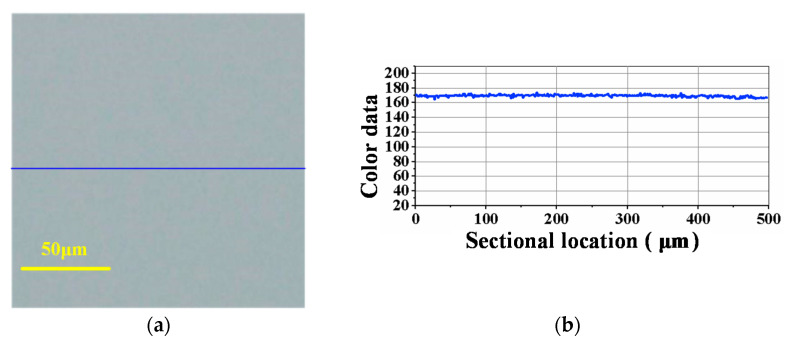
Optical image and sectional surface profile after ultrasonic-assisted CMP using polyurethane pad (1.44 μm/h): (**a**) Surface defects; (**b**) Color data of the sectional profile.

Figure 8 is the surface condition of SiC before lapping, and Figure 9, Figure 10, Figure 11 are the experimental results under the corresponding process in the manufacturing route. From Figure 8, Figure 9, Figure 10, Figure 11, Figure 8a, Figure 9a, Figure 10a and Figure 11a are the surface defects under the optical microscope, Figure 8b, Figure 9b, Figure 10b and Figure 11b are the color data of the sectional surface profile, which can quantitatively reflect the surface roughness. Received SiC wafers were cut from crystal rods by diamond wire saws. The original surface has sawing marks and large brittle pits. The color data of the sectional surface fluctuates greatly, implying that the roughness is poor. After the first step of lapping, the saw marks were removed and the brittle pits were slightly reduced. Accordingly, the fluctuation in the color data was slightly reduced. According to Equation (3), the material removal rate was calculated to be 10.93 μm/min. The results show that ultrasonic lapping using cast an iron disc can quickly remove the saw marks and large surface damages caused by diamond wire-cutting. After the second step of lapping with the MRR of 6.11 μm/min, the surface quality was improved significantly. The number of the residual brittle pits decreased, and the fluctuation in color data curve decreased further. The results show that ultrasonic lapping using a copper disc can remove the residual surface defects at a fast rate. After the third step, namely ultrasonic-assisted CMP, the surface was significantly improved and almost no defects were visible. The fluctuation of sectional surface color data was very small, thereby the curve was basically a straight line. This suggests that the surface roughness of single-crystal SiC is rather low. An ideal surface was formed after the ultrasonic CMP. The material removal rate was calculated to be 1.44 μm/h, which is much higher than the 100 nm/h of traditional CMP. These results show that the ultrasonic-assisted hybrid process can significantly improve the precise machinability of single-crystal SiC, and can hopefully achieve high-efficiency and ultraprecise machining.

## 5. Conclusions

To improve the precise machinability of the single-crystal SiC, this paper proposed an ultrasonic-assisted hybrid machining route. Combining ultrasonic-assisted lapping with ultrasonic-assisted CMP, experiments considering the effects of ultrasonic vibrated amplitude and lapping disc materials on lapping and CMP were performed. Finally, a three-step ultrasonic-assisted precise machining for single-crystal SiC was carried out. Based on the experiments results, the following conclusions can be drawn.

- The established ultrasonic vibration-assisted device can cause the workpiece to longitudinally vibrate with an amplitude from 0 μm to about 3.5 μm.
- Increasing amplitude can increase the processing efficiency of SiC lapping. When the amplitude increases to 2 μm or a higher value, the surface defects and damages can be reduced.
- Ultrasonic-assisted lapping with a proper amplitude can comprehensively improve machining efficiency and surface quality. Less surface damages are beneficial for reducing the subsequent process time. Therefore, ultrasonic assistance promotes the achievement of a finishing surface with a higher MRR.
- Ultrasonic vibration can improve the machining efficiency surface quality of SiC CMP. Due to a higher MRR, the surface defects and damages produced by the former lapping can be quickly removed. Compared with the MRR of 0.347 μm/h in traditional CMP, ultrasonic CMP can achieve a higher MRR of 0.717 μm/h, thus leading to a good surface with less defects.
- The three-step hybrid process, which combines ultrasonic vibration with lapping disc material, can achieve the finishing machining by improving the machining efficiency of lapping and CMP. Ultrasonic lapping using a cast iron disc can quickly remove large surface damages with a high MRR of 10.93 μm/min. Ultrasonic lapping using a copper disc can reduce the residual surface defects with a high MRR of 6.11 μm/min. Ultrasonic CMP can achieve a smooth and less damaged surface, with an MRR of 1.44 μm/h, which is much higher than the 100 nm/h of traditional CMP.
- Thus, this new hybrid process utilizing ultrasonic vibration can significantly improve the precise machinability of single-crystal SiC, and can hopefully achieve high-efficiency and ultraprecise machining.

## Figures and Tables

**Table 1:** The actual values compared with designed value under different powers.

Ultrasonic Power (×2 kW)	Frequency (kHz)	Actual Value (μm)	Designed Output (Input × 0.35 μm)	Input (μm)
40%	19.84	0.5	0.35~0.7	1~2
50%	19.79	0.77	0.7~1.4	2~4
60%	19.75	1.1	1.4	4
70%	19.73	2	2.1	6
80%	19.72	2.6	2.8	8
90%	19.71	3.2	3.5	10

**Table 2:** Ultrasonic-assisted lapping conditions.

P	n	D	t	v	C
40 kPa	50 rpm	4 μm	4 min	12.5 mL/min	2 wt%

**Table 3:** The main machining conditions in the ultrasonic hybrid processes for SiC.

Conditions	Step 1	Step 2	Step 3
Ultrasonic power (×2 kW)	70%	70%	70%
Pressure (kPa)	40	40	60
Rotational speed (rpm)	50	50	50
Machining time (min)	2	4	60
Abrasive size (μm)	6	4	0.08
Abrasive concentration (wt%)	4	4	20
Slurry supplying speed (mL/min)	12.5	12.5	25

## Data Availability

The data presented in this study are available on request from the corresponding author.
